# Crafting chirality in three dimensions via a novel fabrication technique for bound states in the continuum metasurfaces

**DOI:** 10.1038/s41377-023-01368-z

**Published:** 2024-02-05

**Authors:** Zaid Haddadin, Anna My Nguyen, Lisa V. Poulikakos

**Affiliations:** 1 Department of Electrical & Computer Engineering, University of California San Diego, La Jolla, CA USA; 2 Department of Mechanical & Aerospace Engineering, University of California San Diego, La Jolla, CA USA; 3 Program of Materials Science & Engineering, University of California San Diego, La Jolla, CA USA

**Keywords:** Lithography, Metamaterials

## Abstract

An additional deposition step was added to a multi-step electron beam lithographic fabrication process to unlock the height dimension as an accessible parameter for resonators comprising unit cells of quasi-bound states in the continuum metasurfaces, which is essential for the geometric design of intrinsically chiral structures.

Circularly polarised light possesses chirality, i.e., tracing the light path reveals a structure with a mirror image that is not superimposable through rotation or translation operations^1,2^. This distinctiveness of the structure and its mirror image allows for the arbitrary yet specific assignment of left- or right-handedness^1,2^. Illuminating a chiral probe with circularly polarised light results in differential light-matter interactions depending on whether the light is left- or right-handed^1,2^. Manipulating the geometric design of the chiral probe can further tailor these selective light-matter interactions^1,2^.

One technology that can be designed to exhibit chiral optical properties is a metasurface^2^. Metasurfaces are engineered arrangements of subwavelength resonators that can provide tuneable systems to control the interaction of different polarisation states of light with matter^2^. These resonators can be made from different materials—plasmonic^3^, dielectric^4–6^, or a combination of both^7^. To address the high optical losses associated with plasmonic materials, research in metasurfaces has shifted towards all-dielectric material systems^3,5^.

Within this realm of dielectric metasurfaces, the phenomena of bound states in the continuum (BICs) and quasi-bound states in the continuum (qBICs) have been demonstrated^7–9^. BICs are discrete energy states trapped in a system surrounded by a continuum of energy states^7–9^. In contrast, qBICs approximate BICs but allow the release of the trapped discrete energy^7–9^. The intentional design of the resonators enables control over the release of energy in qBIC metasurfaces^7–9^. Transforming a BIC system to a qBIC system necessitates breaking the symmetry of the resonator geometry^10–12^, the resonator arrangement^13^, or the incidence angle of light^10^.

However, most qBIC metasurfaces realized by breaking the symmetry of resonator geometry are constrained to two-dimensional manipulations (Fig. 1a), a consequence of the limitations of fabrication techniques available for all-dielectric metasurfaces^5,10,14–16^. All fabrication techniques must build resonators that are smaller than the operational wavelength^17^. For visible wavelengths, the fabrication techniques can be categorized into lithographical methods, laser methods, or chemical methods^17,18^. Electron beam lithography, used for the majority of reported all-dielectric metasurfaces^17^, offers precision, reliability, and repeatability, but it is limited to two-dimensional elements^16–18^. This drawback hinders the manipulation of the three-dimensional geometry of resonators, which is crucial for the design of maximally chiral probes^19,20^. Consequently, this restricts applications in the study of chirality, including but not limited to fields of analytical chemistry^10–12^, pharmaceutics^6,10^, and the extraterrestrial search for life^6,10,21^.

**Fig. 1:**
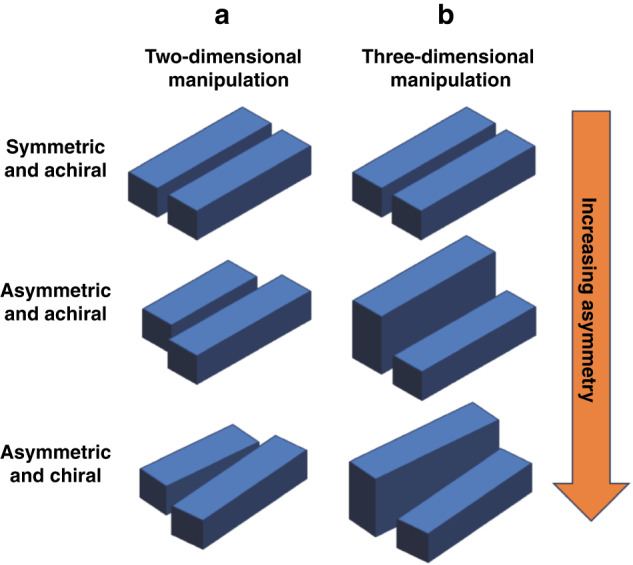
BIC to qBIC by breaking the symmetry of resonator geometry. **a** Two-dimensional and **b** three-dimensional geometric manipulations of anti-parallel rods that can make up the unit cell of a qBIC metasurface. (Top) Symmetric, achiral rods. (Middle) Through resonator symmetry-breaking, the rods comprise an asymmetric unit cell. (Bottom) By tilting the rods towards one another, the unit cell becomes chiral

In a recent publication by Kühner and Wendisch et al. in *Light: Science & Applications*, the research team presented an additional deposition step to a multi-step electron beam lithography fabrication process^5^. This novel nanofabrication methodology provided control over the heights of individual resonators within unit cells comprising all-dielectric metasurfaces^5^. Employing a unit cell composed of two anti-parallel rods (Fig. 1b, Top), the study introduced height disparities between the rods to convert an achiral BIC metasurface into an achiral qBIC metasurface (Fig. 1b, Middle). By tilting the rods of varying heights toward each other, the achiral qBIC metasurface was transformed into a chiral qBIC metasurface (Fig. 1b, Bottom). Continued adjustments to the height difference and angular orientation of the two rods tuned the differential interactions of the chiral qBIC metasurface when illuminated by left- or right-handed circularly polarised light. The final parameters selected yielded a 70% difference in transmittance signals between the two polarisation states of light, underscoring the potential for achieving maximum optical chirality—wherein information from one handedness of light–matter interactions cannot be obtained from the opposite handedness, i.e., a 100% difference in signals^22^.

This work introduced a new level of fabrication complexity, offering a previously unattainable degree of freedom for tailoring the optical response of chiral metasurfaces by unlocking the height dimension of resonators for geometric manipulation^5^. Further efforts to expand this freedom to the Angstrom level could pave the way for maximum chirality in response to electromagnetic waves from arbitrary angles of incidence because such small resolutions may permit the systematic study of the asymmetry of all reflection and transmission processes^5,6,19,22–24^. Nonetheless, these results hold promise for chiral nanophotonic applications in biochemical sensing^25^, enantiomeric separation^11,12^, polarisation conversion^13^, and chiral emission^26^.
